# Fibroblast Growth Factor 23-Producing Phosphaturic Mesenchymal Tumor with Extraordinary Morphology Causing Oncogenic Osteomalacia

**DOI:** 10.3390/medicina56010034

**Published:** 2020-01-16

**Authors:** Cornelia Then, Evelyn Asbach, Harald Bartsch, Niklas Thon, Christian Betz, Martin Reincke, Ralf Schmidmaier

**Affiliations:** 1 Department of Endocrinology, Medizinische Klinik und Poliklinik IV, Ludwig-Maximilians-University, 80336 Munich, Germany; 2 Institute of Pathology, Klinikum der Universität München, Ludwig-Maximilians-University, 80337 Munich, Germany; 3 Department of Neurosurgery, Klinikum Universität München, Ludwig-Maximilians-University, 81377 Munich, Germany; 4 Department of Otorhinolaryngology, University Medical Center Hamburg-Eppendorf, 20246 Hamburg, Germany

**Keywords:** FGF-23, phosphaturic mesenchymal tumor, PMT, hypophosphatemia

## Abstract

A possible cause of hypophosphatemia is paraneoplastic secretion of fibroblast growth factor 23 (FGF-23). Tumors secreting FGF-23 are rare, mostly of mesenchymal origin, usually benign, and may be located anywhere in the body, including hands and feet, which are often not represented in conventional imaging. A 50-year-old woman presented with diffuse musculoskeletal pain and several fractures. Secondary causes of osteoporosis were excluded. Laboratory analysis revealed hypophosphatemia and elevated alkaline phosphatase, parathyroid hormone, and FGF-23. Thus, oncogenic osteomalacia due to neoplastic FGF-23 secretion was suspected. FDG-PET-CT and DOTATATE-PET-CT imaging demonstrated no tumor. Cranial MRI revealed a tumorous mass in the left cellulae ethmoidales. The tumor was resected and histopathological examination showed a cell-rich tumor with round to ovoid nuclei, sparse cytoplasm, and sparse matrix, resembling an olfactory neuroblastoma. Immunohistochemical analysis first led to diagnosis of olfactory neuroblastoma, which was later revised to phosphaturic mesenchymal tumor. Following the resection, FGF-23 and phosphate levels normalized. In conclusion, we here describe a patient with an FGF-23-secreting phosphaturic mesenchymal tumor with an unusual morphology. Furthermore, we emphasize diagnostic pitfalls when dealing with FGF-23-induced hypophosphatemia.

## 1. Introduction

Hypophosphatemia has several possible causes including reduced intestinal absorption (i.e., steatorrhea, treatment with antacids), shifting from the extracellular to the intracellular department (i.e., refeeding, alcalosis), and increased renal losses. The phosphatonine fibroblast growth factor 23 (FGF-23) leads to renal phosphate loss through inhibition of the sodium–phosphate cotransporter in the proximal tubule. FGF-23 further blocks 25-OH-vitamin D_3_ hydroxylation via inhibition of the 1-alpha-hydroxylase. It is mainly synthetized in osteoblasts and rarely in mesenchymal tumors, which can be located in all body parts [1]. Elevated FGF-23 levels may also be present in hereditary forms of hypophosphatemia (e.g., X-linked hypophosphatemic rickets). However, hereditary osteomalacia usually manifests during early age. Adult patients presenting with hypophosphatemia require a thorough diagnostic work-up including the search for an FGF-23-secreting mesenchymal tissue tumor [2].

## 2. Case Report and Results

A 50-year-old woman (written consent was obtained from the patient and the faculty ethics committee (20-047KB)) presented in our clinic in December 2015 with diffuse bone, muscle, and joint pain. She reported the first symptoms about a year earlier and an aggravation ever since. During that time, she had consulted several outpatient physicians. Dual energy X-ray absorptiometry had shown osteopenia with a minimal T-score of −2.0 standard deviations at the lumbar spine. A skeletal scintigraphy had displayed nuclear activity enrichments in the right ribs, thoracic spine, hips, and facial bones. Menopause started in 2014 and the patient received hormone replacement therapy for six months before the presentation in our clinic. Further therapy included cholecalciferol and intravenous bisphosphonates, the latter under the assumption of a transitory osteoporosis of the left hip. Secondary causes of osteoporosis (i.e., hypercortisolism, mastocytosis, monoclonal gammopathy, rheumatological disorder, gynaecological tumor or other malignancy, Paget’s disease) had been excluded. Since no plausible explanation had been found for the findings and symptoms, the complaints had been interpreted as a consequence of degenerative spinal changes.

The patient eventually presented in our clinic with new pain in the right knee and left foot. MRI imaging revealed a fracture of the first left metatarsal bone with concomitant oedema, oedema without fracture in the spongiosa of the medial talus and the os cuneiforme laterale, as well as infractions in the right inferior patella epiphysis and the anteromedial tibia. In the short run, the patient developed progressive pain in both knees and became unable to walk. The MRI scan now displayed ostechondrosis dissecans of the left medial talus plus various fractures (medial metaphysis of the right tibia head, medial femur condyles, distal cuboid and proximal os cuneiforme). The patient denied an adequate trauma or maltreatment. 

Laboratory analysis revealed normocalcemia, but considerable hypophosphatemia (1.6 mg/dL; range: 2.5–4.8 mg/dL) and a moderately elevated alkaline phosphatase (114 U/L; range: 35–105 U/L) and parathyroid hormone (185 pg/mL; range: 15–65 pg/mL). Retrospective resurvey of external laboratory values also showed decreased phosphate levels, albeit not consistently that distinct. The 25-OH-vitamin D_3_ level was normal (39 ng/mL; range 20–60 ng/mL), whereas 1,25-OH-vitamin D_3_ was decreased (13 ng/L; range 25–86 ng/L). Urinary analysis displayed a low phosphate value (17.9 mg/dL; reference 22–74 mg/dL). However, fractional phosphate excretion (48%; reference 5–20%) and phosphate clearance (44 mL/min; reference 5.4–16.2 mL/min) were increased. C-terminal FGF-23 was elevated (224 kRU/L; reference 26–111 kRU/L). 

**Figure 1:**
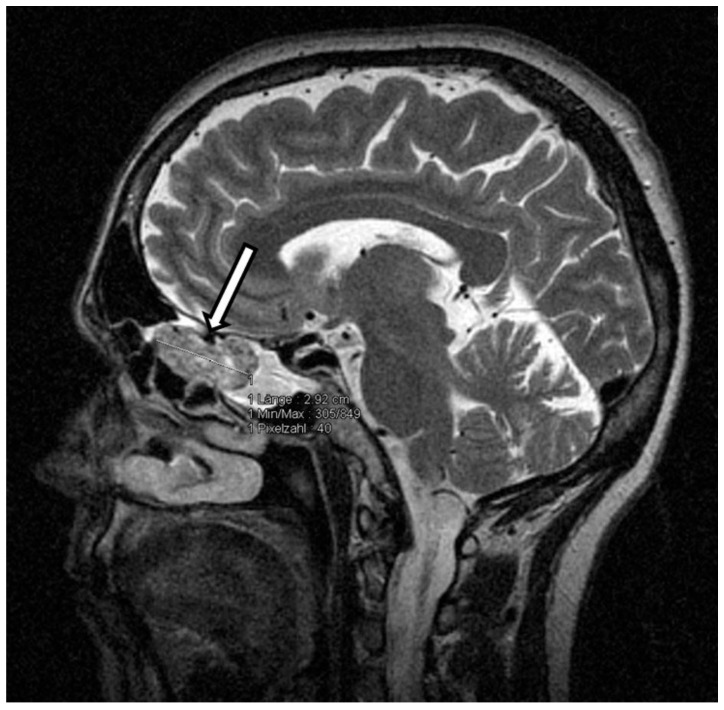
MRT scan of the cranium. Arrow—tumorous mass in the left cellulae ethmoidales.

Thus, oncogenic osteomalacia due to neoplastic FGF-23 secretion was suspected. FDG-PET-CT and subsequently DOTATATE-PET-CT imaging demonstrated no tumor. Since the cranium was not represented in both PET-CT scans, contrast enhanced cranial MRI was performed and finally revealed a tumorous mass in the left cellulae ethmoidales with an expansion of 3.2 cm × 1.4 cm × 1.8 cm and intensive contrast enhancement (Figure 1). Remarkably, retrospective analysis of an MRI scan of the head, which had been performed months earlier in an outpatient clinic, already showed the tumor, which had not been described back then. Even in retrospect, our patient denied symptoms possibly related to a tumor located in the cellulae ethmoidales, such as nasal obstruction, epistaxis, hyposmia, diplopia, tuba eustachii obstruction, or frontal headache. 

**Figure 2:**
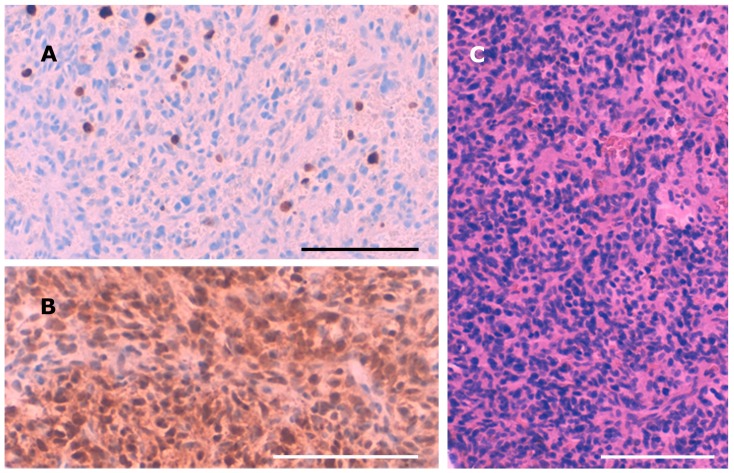
Histopathological examination revealed a cell-rich but poorly proliferating (Ki67: 5% (**A**)), neuron-specific, enolase-positive (**B**) PMT. (**C**) Hematoxylin-eosin stain. Bar = 100 µm.

Intraoperatively, the tumor presented as a bleeding, amorphous mass with bony erosion of the posterior ethmoidal cells. It was fully resected in piecemeal technique via expanded endonasal surgery including the left cribriform plate as well as the left papyraceous membrane. The skull base defect was successfully closed using a pedicled nasoseptal flap. Histopathological examination showed a neuron-specific-enolase-positive, cell-rich but poorly proliferating (Ki67: 5%) tumor (Figure 2). The nuclei were moderately pleomorphic. Pseudorosettes were found in some tumor areas. No necrotic areas were observed. Staining for CD3, CD20, CD45, GFAP, CD31, S100, STAT6, actin, HMB45, CD99, EMA, TLE, synaptophysin, chromogranin, CD34, CD68, KL1, desmin, and ceratin was negative. Only scarce tumor matrix was observed, while hyalinized matrix would be a leading feature for the diagnosis of a phosphaturic mesenchymal tumor (PMT). The tumor showed a well-developed capillary network, though perocytoma-like pattern or haemangioma-like pattern was not observed. Calcification was not seen. The location in the cellulae ethmoidales and the histopathological features of the tumor first led to the diagnosis of an olfactory neuroblastoma. However, additional staining for SATB2 and beta-Catenin revealed nuclear positivity for SATB2 and nuclear negativity for beta-Catenin, confirming the diagnosis of PMT.

**Figure 3:**
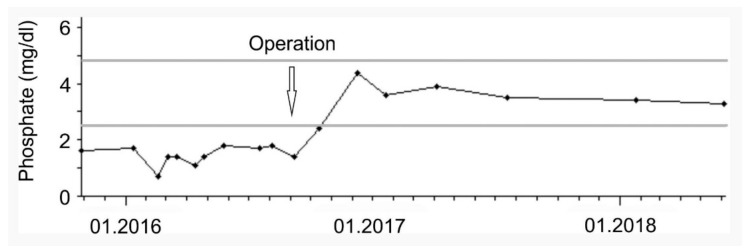
Serum phosphate levels of the described patient over time.

Following the resection, FGF-23 (63 kRU/L), parathyroid hormone, and phosphate levels (Figure 3) normalized and phosphate supplementation was terminated. The patient described a significant improvement of her painful symptoms and general condition and regained the ability to walk and participate in daily life activities without restrictions. 

## 3. Discussion

PMTs are rare mesenchymal tumors that are claimed to be morphologically distinctive neoplasms [3]. Due to the rarity of the disease, the prevalence of oncogenic osteomalacia is not known. The literature and Orphanet report approximately 400 cases worldwide to date [1,4]. Most tumors occur in middle-aged adults. Often, patients are diagnosed late, with a long history of osteomalacia, which was also true for our patient. The reported case demonstrates several difficulties that can occur when dealing with an orphan disease. Hypophosphatemia had been documented months before the final diagnosis was established. Since calcium levels had been normal and parathyroid hormone levels were only inconsistently elevated, too little attention was paid to this single laboratory parameter, which is often only attended in context with changes in calcium levels or when being elevated in chronic kidney disease. Further, once suspected, localizing a PMT may be challenging. PMTs are typically benign, slowly growing, and therefore often small and without local symptoms. Furthermore, they do not have a predilection site, but may occur anywhere in the body, including unexpected locations, such as hands and soles of the feet [5]. In our case, the tumor itself had been pictured months before the final diagnosis, but was not identified as such, probably because the MRI scan was performed in order to detect skeletal changes and a soft tissue tumor was not expected. Further, although we were aware of the necessity of a whole-body scan when searching for an FGF-23-producing tumor and also communicated this to our radiology department, the cranium was not pictured in both performed PET-CT scans. Finally, the unusual location and morphology of the tumor led to the initial diagnosis of an olfactory neuroblastoma that was later revised to PMT after additional immunohistochemical analyses. 

## 4. Conclusions

The reported case emphasizes the importance of sufficient diagnostic procedures and their adequate interpretation in rare diseases in order to avoid unnecessary costs and intolerable time losses. The present case shows that even isolated and possibly at first glance inconspicuous anomalies require further evaluation and possibly referral to a specialized center. Furthermore, it has to be ensured that all persons participating in the diagnostic procedures are familiar with (known) diagnostic difficulties in rare diseases, such as the challenging tumor localization in suspected FGF-23-producing tumors. Last, inconsistencies (such as the assumption of an FGF-23-producing olfactory neuroblastoma, which has not been described before) should trigger a deeper investigation, since in the presented case the treatment would have differed considerably if the diagnosis of a neuroblastoma had been kept.

## 5. Limitations

We did not analyze other phosphaturic proteins in our patient, since FGF-23 was elevated, and phosphate as well as FGF-23 returned to normal levels after surgery. We therefore assume that FGF-23 was the causative agent for the hypophosphatemia. However, we cannot exclude that the tumor may have coexpressed other phosphaturic proteins, such as FGF7, matrix extracellular phosphoglycoprotein, and secreted frizzled-related protein 4.
